# Fatty Acid Synthase Inhibitor G28 Shows Anticancer Activity in EGFR Tyrosine Kinase Inhibitor Resistant Lung Adenocarcinoma Models

**DOI:** 10.3390/cancers12051283

**Published:** 2020-05-19

**Authors:** Emma Polonio-Alcalá, Sònia Palomeras, Daniel Torres-Oteros, Joana Relat, Marta Planas, Lidia Feliu, Joaquim Ciurana, Santiago Ruiz-Martínez, Teresa Puig

**Affiliations:** 1 New Therapeutic Targets Laboratory (TargetsLab)-Oncology Unit, Department of Medical Sciences, Faculty of Medicine, University of Girona, 17003 Girona, Spain; emma.polonio@udg.edu (E.P.-A.); sonia.palomeras@udg.edu (S.P.); 2 Product, Process and Production Engineering Research Group (GREP), Department of Mechanical Engineering and Industrial Construction, University of Girona, 17003 Girona, Spain; quim.ciurana@udg.edu; 3 Department of Nutrition, Food Sciences and Gastronomy, School of Pharmacy and Food Sciences, Food Torribera Campus, University of Barcelona, E-08921 Santa Coloma de Gramanet, Spain; danytoot@hotmail.com (D.T.-O.); jrelat@ub.edu (J.R.); 4 Institute of Nutrition and Food Safety of the University of Barcelona (INSA-UB), E-08921 Santa Coloma de Gramenet, Spain; 5 CIBER Physiopathology of Obesity and Nutrition (CIBER-OBN), Instituto de Salud Carlos III, E-28029 Madrid, Spain; 6 LIPPSO, Department of Chemistry, University of Girona, 17003 Girona, Spain; marta.planas@udg.edu (M.P.); lidia.feliu@udg.edu (L.F.)

**Keywords:** NSCLC, EGFR TKI, FASN inhibitors, resistance, STAT3, EGCG

## Abstract

Epidermal growth factor receptor (EGFR) tyrosine kinases inhibitors (TKIs) are effective therapies for non-small cell lung cancer (NSCLC) patients whose tumors harbor an EGFR activating mutation. However, this treatment is not curative due to primary and secondary resistance such as T790M mutation in exon 20. Recently, activation of transducer and activator of transcription 3 (STAT3) in NSCLC appeared as an alternative resistance mechanism allowing cancer cells to elude the EGFR signaling. Overexpression of fatty acid synthase (FASN), a multifunctional enzyme essential for endogenous lipogenesis, has been related to resistance and the regulation of the EGFR/Jak2/STAT signaling pathways. Using EGFR mutated (EGFRm) NSCLC sensitive and EGFR TKIs’ resistant models (Gefitinib Resistant, GR) we studied the role of the natural polyphenolic anti-FASN compound (−)-epigallocatechin-3-gallate (EGCG), and its derivative G28 to overcome EGFR TKIs’ resistance. We show that G28’s cytotoxicity is independent of TKIs’ resistance mechanisms displaying synergistic effects in combination with gefitinib and osimertinib in the resistant T790M negative (T790M−) model and showing a reduction of activated EGFR and STAT3 in T790M positive (T790M+) models. Our results provide the bases for further investigation of G28 in combination with TKIs to overcome the EGFR TKI resistance in NSCLC.

## 1. Introduction

Lung cancer is the most incident and the leading cause of cancer death worldwide. Non-small cell lung cancer (NSCLC) subtype is the most common and it represents 80–85% of lung cancers diagnosed. Early-stage NSCLC patients have long-term survival after surgery. However, approximately 75% of patients are diagnosed in advanced stages [1,2].

Gefitinib is a first generation epidermal growth factor receptor (EGFR) tyrosine kinase inhibitor (TKI). It was approved in 2003 by the Food and Drug Administration (FDA) for the treatment of patients whose tumors harbor an EGFR sensitizing/activating mutation (EGFRm) i.e., exon 19 deletion (ΔE746-A750) or the point mutation in exon 21 that leads to the amino acid substitution L858R [3,4,5]. Despite this therapy represents a breakthrough in the treatment of EGFRm NSCLC patients, in a median time of 9–16 months nearly all patients do not achieve a complete response. One of the most common resistance mechanisms described is the EGFR point mutation in exon 20 that leads to the replacement of threonine 790 by methionine (T790M), which normally derives to lethal disease progression [6]. Osimertinib is an irreversible third generation TKI effective in EGFRm T790M positive (T790M+) patients. However, the point mutation C797S in exon 20 has appeared as the main resistance mechanism to the latest FDA-approved TKI [6]. Other mechanisms for EGFR TKI resistance include Met amplification, phosphatidylinositol-4,5-bisphosphate 3-kinase catalytic subunit alpha (PI3KCA) mutations, appearance of stem-like properties as evidenced by increase in epithelial–mesenchymal transition (EMT) and histological transformation, epidermal growth factor receptor type 2 (ErbB2) gene amplification, increase of insulin-like growth factor 1 receptor (IGF-1R) signaling pathway through the loss of inhibitory insulin-like growth factor-binding protein (IGF-BP) and loss or reduction of phosphatase and tensin homolog (PTEN), activation of AXL tyrosine kinase receptor or B-Raf proto-oncogene, and serine/threonine kinase (BRAF) mutations [6,7,8,9,10,11,12].

Recently, the activation of a signal transducer and activator of transcription 3 (STAT3) has been described as an alternative resistance mechanism allowing cancer cells to escape the EGFR signaling or the TKI suppression [13]. STAT3 is involved in the transcription of many genes related to cell differentiation, proliferation, resistance to apoptosis, angiogenesis, metastasis, and immune response [14,15,16]. Besides being phosphorylated by EGFR [17], STAT3 can also be activated in response to different cytokines and growth factors such as interleukin 6 (IL-6), interferon-alpha (IFN-α) or epidermal growth factor (EGF), among others [18]. Approximately 60% of patients show STAT3 activation, which correlates with poorly differentiated tumors, the presence of metastasis, and the late clinical stage [19,20]. STAT3 phosphorylation has been related to disease progression in a small cohort of patients after EGFR TKI treatment [21]. Additionally, neither gefitinib nor osimertinib are able to inhibit STAT3 activation [22,23].

Energy metabolism deregulation has been described as a hallmark of cancer, allowing cell growth and proliferation [24]. Fatty acid synthase (FASN) is an essential enzyme for the de novo synthesis of long-chain fatty acids from acetyl-CoA, malonyl-CoA, and NADPH [25]. Unlike most normal cells, highly-proliferative cancer cells overexpress this lipogenic enzyme, being an interesting target in cancer therapy [26,27]. FASN is strongly associated to poor prognosis and resistance to treatment in different human tumors such as breast [28], bladder [29], pancreatic [30], or lung cancer [31]. Moreover, FASN overexpression has also been proposed as a multidrug resistance mechanism, protecting cells from drug-induced apoptosis through the overproduction of palmitic acid [32]. The natural compound (−)-epigallocatechin-3-gallate (EGCG) is a polyphenolic flavonoid from green tea that has been broadly studied for its cardiovascular, neuroprotective, anticancer, and antimicrobial properties [33,34]. EGCG has been reported to compete with NADPH to bind the β-ketoacyl reductase domain of FASN [35]. The ability of several FASN inhibitors to regulate the canonical EGFR/Jak2/STAT pathway has also been stated in the literature [36,37]. We and others have shown that FASN inhibition is mainly related to EGFR/HER2 signaling pathways, leading to cytotoxic effects in vitro and in vivo in a wide range of carcinomas, including breast and lung [38,39,40,41,42]. To date, many EGCG derivatives have been developed to improve efficacy and increase stability in physiological conditions. Among them, the naphthalene derivative G28 has shown interesting antiproliferative features against sensitive and resistant breast cancer cells [38,43,44].

The purpose of this work was to study the role of FASN inhibitors (EGCG and G28) to overcome TKI resistance in NSCLC. FASN inhibitors were tested alone and in combination with EGFR TKIs (gefitinib and osimertinib) in EGFRm NSCLC models resistant to EGFR TKIs (Gefitinib Resistant, GR). In addition, we also evaluated gene and protein expression changes of FASN, EGFR, and STAT3 resulting from treatments with FASN inhibitors and EGFR TKIs alone or in combination. We show that FASN inhibitor G28 cytotoxicity is independent of EGFR TKI resistance mechanisms. Interestingly, G28 compound exhibited a cytotoxic effect in combination with gefitinib showing changes in EGFR/STAT3 pathway in T790M positive (T790M+) GR models and strong synergism in combination with gefitinib or osimertinib in T790M negative (T790M−) GR model.

## 2. Results

### 2.1. EGFRm GR NSCLC Models Are Sensitive to FASN Inhibition

In order to study the role of FASN in the acquisition of EGFR TKI resistance in NSCLC, we used the sensitive PC9 cell line carrying the EGFR exon 19 deletion (ELREA) and three GR models, two T790M+ models (PC9-GR1 and PC9-GR4), and one T790M− model (PC9-GR3) [45].

#### 2.1.1. EGFRm NSCLC Models Overexpress FASN

**Figure 1:**
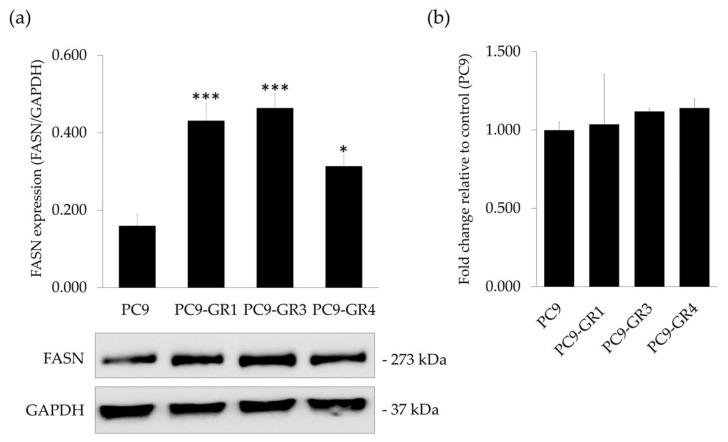
FASN protein and mRNA expression levels in sensitive (PC9) and Gefitinib Resistant (GR) models (PC9-GR1, PC9-GR3, and PC9-GR4). (**a**) Western blot analysis (quantification in upper panel and bands in lower panel) of FASN protein expression. GAPDH was used as a loading control. Results shown are representative from at least three independent experiments. (**b**) FASN endogenous mRNA levels were obtained by qRT-PCR and normalized against the GAPDH gene. FASN expression in the sensitive cells was normalized to 1 an expressed as a fold change, to which all other conditions were compared. Results shown are mean ± SE from three independent experiments. * *p* < 0.050, *** *p* < 0.001 indicate levels of statistically significance.

Firstly, we determined whether EGFRm NSCLC models express FASN enzyme. Hence, FASN protein (Figure 1a) and mRNA expression levels (Figure 1b) were analyzed by immunoblotting and quantitative real time PCR (qRT-PCR), respectively.

All models showed FASN protein and mRNA expression. Despite no differences in mRNA, GR models presented significantly higher protein expression levels (PC9-GR1 *p* = 8.710 × 10^−4^; PC9-GR3 *p* = 3.160 × 10^−4^, and PC9-GR4 *p* = 0.049) in comparison to PC9.

#### 2.1.2. PC9-GR3 Model Is Resistant to Gefitinib and Osimertinib

**Figure 2:**
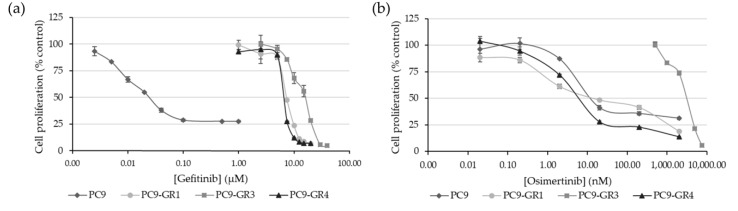
Cell proliferation inhibition of EGFR TKIs (gefitinib and osimertinib) in parental and Gefitinib Resistant (GR) models. Sensitive (PC9) and GR models (PC9-GR1, PC9-GR3, and PC9-GR4) were treated with increasing concentrations of (**a**) gefitinib (from 2.5 × 10^−3^ to 1 µM for PC9 and 1–40 µM for GR models) and (**b**) osimertinib (0.02–2000 nM for PC9, PC9-GR1, and PC9-GR4 and 500–7500 nM for PC9-GR3) for 72 h. Results shown are expressed as percentage of surviving cells after drug treatment (mean ± SE) and are representative from at least three independent experiments.

We confirmed the resistance to EGFR TKIs in PC9 and GR models. For that, we measured the cytotoxic effect of gefitinib and osimertinib on all models by determining the half-maximal inhibitory concentration (IC_50_) using the MTT assay (Figure 2).

As expected, GR models were significantly more resistant to gefitinib with IC_50_ values in the micromolar range compared to the nanomolar IC_50_ found in the PC9 cell line (PC9-GR1 *p* = 2.793 × 10^−7^; PC9-GR3 *p* = 1.631 × 10^−10^, and PC9-GR4 *p* = 1.000 × 10^−6^). Although no significant differences were found in the IC_50_ value for gefitinib between the two T790M+ GR models, the IC_50_ value of the PC9-GR3 model for gefitinib was significantly greater than PC9-GR1 (*p* = 7.953 × 10^−7^) and PC9-GR4 (*p* = 1.659 × 10^−7^). PC9-GR3 model was also resistant to osimertinib compared to other models (PC9 *p* = 2.799 × 10^−9^; PC9-GR1 *p* = 3.749 × 10^−8^, and PC9-GR4 *p* = 5.200 × 10^−9^).

#### 2.1.3. FASN Inhibitors Present Cytotoxic Effects in NSCLC Models

**Figure 3:**
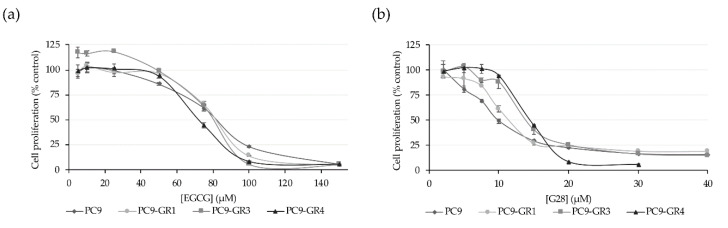
Cell proliferation inhibition of FASN inhibitors in parental and Gefitinib Resistant (GR) models. Sensitive (PC9) and GR models (PC9-GR1, PC9-GR3, and PC9-GR4) were treated with increasing concentrations of (**a**) EGCG (5–150 µM) and (**b**) G28 (2–40 µM) for 72 h. Results shown are expressed as the percentage of surviving cells after drug treatment (mean ± SE) and are representative from at least three independent experiments.

Cancer cells have been described to increase the de novo lipogenesis through the activation of FASN and its inhibition has proven to cause cell death. Therefore, this enzyme has become a promising candidate for the development of new anticancer therapies. Here we tested the cytotoxic activity of the two FASN inhibitors, EGCG and its derivative G28. MTT cell viability assays showed that the natural polyphenolic compound EGCG was cytotoxic for PC9 (IC_50_ = 77.9 ± 1.9 µM), PC9-GR1 (IC_50_ = 74.3 ± 4.3 µM), PC9-GR3 (IC_50_ = 91.0 ± 5.5 µM), and PC9-GR4 (IC_50_ = 75.6 ± 2.4 µM) NSCLC models with no significant differences (*p* = 0.358; Figure 3a).

The synthetic EGCG derivative G28 showed higher cytotoxicity in all tested models with IC_50_ of 12.8 ± 1.3 µM for PC9, 12.0 ± 0.8 µM for PC9-GR1, 17.8 ± 1.3 µM for PC9-GR3, and 11.2 ± 1.2 µM for PC9-GR4 (Figure 3b). Besides, only PC9-GR3 showed a significantly higher IC_50_ value compared to PC9 (*p* = 0.030), PC9-GR1 (*p* = 0.005), and PC9-GR4 (*p* = 0.002).

#### 2.1.4. G28 Inhibits FASN in EGFRm NSCLC Models

**Figure 4:**
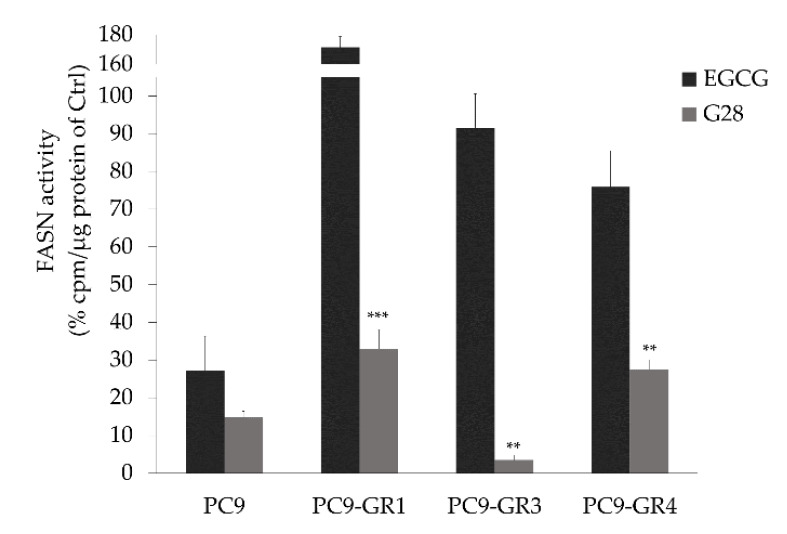
G28 compound inhibits FASN activity in Gefitinib Resistant (GR) models. Cells were treated for 72 h with EGCG or G28 at a concentration equal to their IC_50_ and with DMSO as control. FASN activity was assayed by counting radiolabeled fatty acids synthesized de novo. Bars represent the remaining activity as a percentage in treated versus untreated cells (control, Ctrl). Data are mean ± SE from at least three independent experiments. ** *p* < 0.010, *** *p* < 0.001 indicate levels of statistically significance.

The ability to internalize and inhibit FASN activity of EGCG and G28 after being exogenously added to the media was tested in sensitive and GR models (Figure 4). EGCG and G28 inhibited FASN in PC9 cells, resulting in a similar FASN activity reduction of roughly 80% (*p* = 0.265). Moreover, G28 significantly reduced FASN activity in all GR models in comparison with EGCG (PC9-GR1 *p* = 3.000 × 10^−5^; PC9-GR3 *p* = 0.001; PC9-GR4 *p* = 0.008) while the EGCG compound was not able to diminish FASN activity.

#### 2.1.5. Apoptosis Induction of FASN Inhibitors and EGFR TKIs Treatments

**Figure 5:**
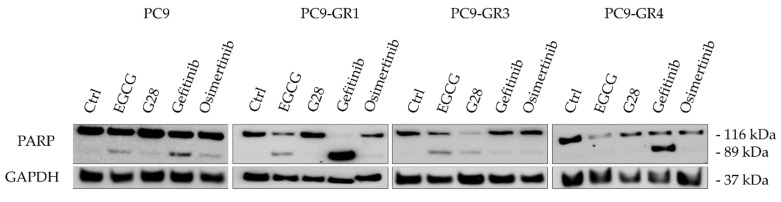
Effects of FASN inhibitors and EGFR TKIs on apoptosis determined by PARP cleavage. Sensitive (PC9) and Gefitinib Resistant (GR) models (PC9-GR1, PC9-GR3, and PC9-GR4) were treated for 72 h with a concentration equivalent to IC_50_ of each drug. Untreated cells were used as an internal control (Ctrl) and GAPDH as a loading control. Results shown are representative from at least three independent experiments.

We also investigated whether the cell death caused by treatment with EGFR TKIs or FASN inhibitors was the result of apoptosis induction in both sensitive and GR models. Poly(ADP-ribose) polymerase (PARP) terminal proapoptotic protein activated after cleavage was used as an apoptosis marker. The effects of all compounds on PARP was determined by Western blot analysis in all models (Figure 5). The uncropped Western blots can be found in Figure S2a.

The cleaved form of PARP (89 kDa) appeared in either sensitive or resistant T790M− models after treatment with the IC_50_ concentration of both EGFR TKIs (gefitinib and osimertinib) and FASN inhibitors (EGCG and G28), indicating the induction of apoptosis. Gefitinib treatment led to PARP cleavage in T790M+ GR models. Additionally, EGCG and osimertinib led to the formation of cleaved PARP in PC9-GR1 model.

### 2.2. G28 Increases EGFR Activation in EGFRm NSCLC Models

**Figure 6:**
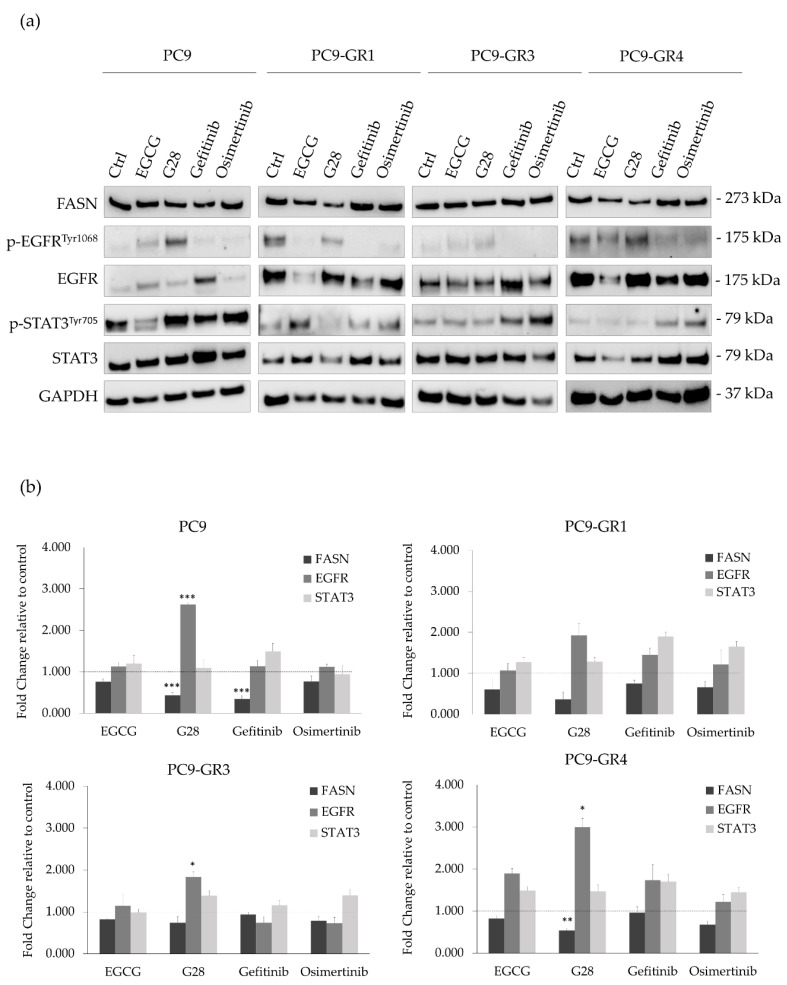
FASN, EGFR, and STAT3 protein and mRNA expression levels after FASN inhibitors (EGCG and G28) and EGFR TKIs (gefitinib and osimertinib) treatment in sensitive (PC9) and Gefitinib Resistant (GR) models (PC9-GR1, PC9-GR3, and PC9-GR4). (**a**) Western blot analysis of FASN, EGFR, and STAT3 protein expression after 72 h of FASN inhibitors (IC_50_) and EGFR TKIs (IC_50_) treatment in EGFR TKI sensitive and GR models. Untreated cells were used as an internal control (Ctrl) and GAPDH as a loading control. Results shown are representative from at least three independent experiments. (**b**) FASN, EGFR, and STAT3 mRNA levels after treatment with FASN inhibitors and EGFR TKIs in sensitive and GR models. mRNA levels were obtained by qRT-PCR and normalized against the GAPDH gene. All conditions were compared to control (untreated cells), which was normalized to 1 (indicated by the dotted line) and expressed as a fold change. Experiments were performed at least three times. * *p* < 0.050, ** *p* < 0.010, *** *p* < 0.001, indicate levels of statistically significance.2.3. G28 Combined with EGFR TKIs Outcomes in Synergistic Effects.

FASN has been previously related to AKT/ERK/EGFR signaling pathways [46] and the inhibition of the transcription factor STAT3 [36] in lung adenocarcinomas. Thus, differences in FASN, EGFR, and STAT3 protein and mRNA expression levels after FASN inhibitors or EGFR TKIs treatment were analyzed through immunoblotting (Figure 6a) and qRT-PCR (Figure 6b) in PC9 and GR models. The uncropped Western blots can be found in Figure S2b.

A reduction of FASN mRNA expression levels was observed in sensitive and GR models treated with FASN inhibitors, being statistically significant in the PC9-GR4 treated with G28 (*p* = 0.004) and PC9 cells treated with G28 (*p* = 7.370 × 10^−4^) and gefitinib (*p* = 3.210 × 10^−4^). T790M+ GR models presented a basal hyperactivation of EGFR that was inhibited after treatment with EGFR TKIs. Regarding FASN inhibitors, EGCG and G28 increased its activation in PC9 cells contrary to the PC9-GR1 model, while no changes were observed in PC9-GR3 and PC9-GR4 models.

EGCG reduced EGFR protein levels in sensitive and T790M+ GR models that did not correlate with mRNA expression levels. Contrary, G28 significantly increased EGFR mRNA expression in all models, without protein level modification. No changes in EGFR mRNA levels were observed after gefitinib treatment, however higher protein levels were detected in T790M− GR models. Osimertinib treatment did not lead to EGFR protein nor mRNA expression alteration. EGFR TKIs treatment increased STAT3 activation in all GR models. Gefitinib increased p-STAT3 levels in sensitive cells, while the highest STAT3 activation in GR models was found with osimertinib treatment. No STAT3 protein or mRNA expression differences were found in any of the models and treatments assayed.

Lung carcinomas are highly proliferative and resistance acquisition after EGFR TKI-based therapy is a major problem. Overproduction of palmitic acid by FASN emerges as a resistance mechanism, protecting cells from drug-induced apoptosis [47]. The combination of these drugs with a different mechanism of action may decrease resistance development and improve treatment response.

**Figure 7:**
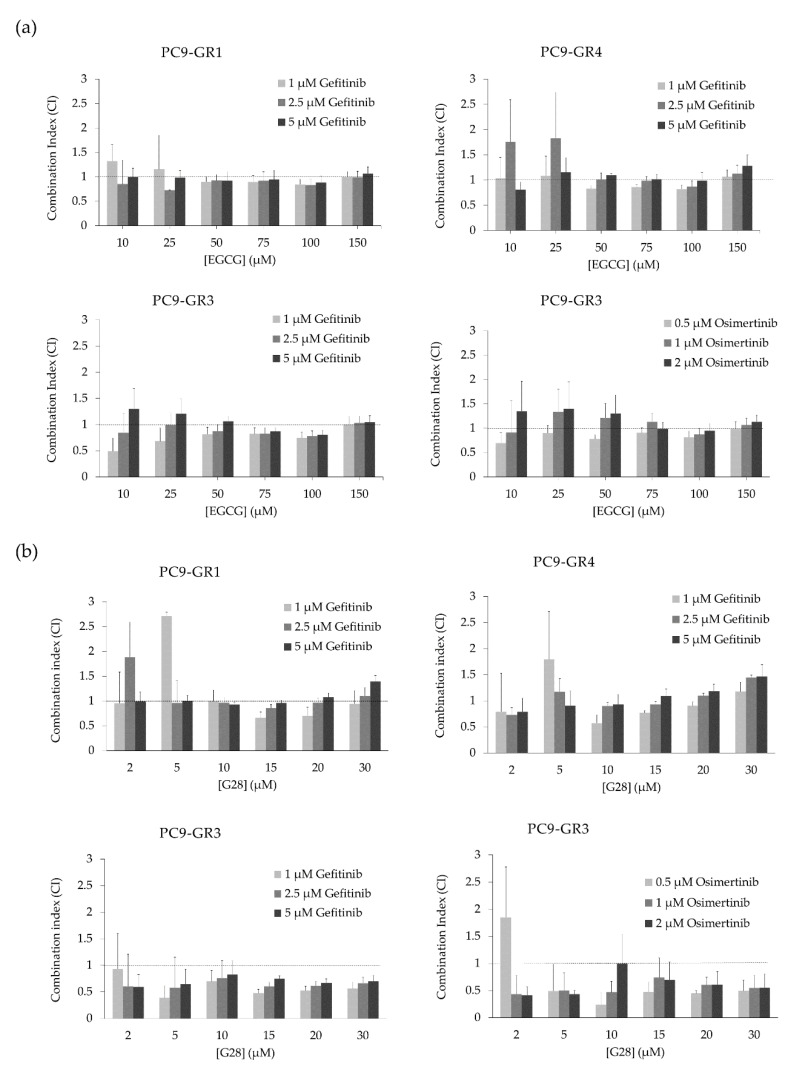
Combination index (CI) of FASN inhibitors (EGCG and G28) and EGFR TKIs (gefitinib and osimertinib) treatment in Gefitinib Resistant (GR) models (PC9-GR1, PC9-GR3, and PC9-GR4). (**a**) CI of EGCG and gefitinib in PC9-GR1, PC9-GR3, and PC9-GR4 models or osimertinib in PC9-GR3. (**b**) CI of G28 and gefitinib in PC9-GR1, PC9-GR3, and PC9-GR4 models or osimertinib in PC9-GR3. PC9-GR1, PC9-GR3, and PC9-GR4 models were treated with EGCG (10–150 µM) or G28 (2-30 µM) in combination with gefitinib (1, 2.5, and 5 µM) for 72 h. PC9-GR3 cells were also treated with EGCG (10–150 µM) or G28 (2–30 µM) in combination with osimertinib (0.5, 1, and 2 µM) for 72 h. Results were determined using the MTT assay and are expressed as the CI based on the Chou and Talalay method [48]. The dotted line indicates additivism (CI approximately equal to 1). CI > 1 designates antagonistic effects and CI < 1 synergistic effects. Experiments were performed at least three times. Results shown are mean ± SE.

Once the effects of FASN inhibitors and EGFR TKIs alone were analyzed in all models, the combinatorial treatment between FASN inhibitors and EGFR TKIs was also studied in order to evaluate the possible synergistic interactions (Figure 7 and Figure S1). Despite the fact that EGCG did not reduce FASN activity in GR models, no IC_50_ differences were found in comparison with sensitive cells. Therefore, GR models were treated with EGCG or G28 in combination with the EGFR TKI to which they were resistant. Three EGFR TKIs concentrations were chosen based on MTT assays (vide supra). The combination of EGCG with either gefitinib or osimertinib resulted in mostly additive effect as shown by the combination index (CI; Figure 7a). The combination of G28 with gefitinib generally led to additivism in T790M+ GR models, with some synergism found in G28 concentrations ranging from 10 to 20 µM. Remarkably, T790M− GR model treated with G28 combined with gefitinib or osimertinib showed greater synergistic effects (Figure 7b).

### 2.3. G28 Reduces STAT3 Activation in T790M+ GR Models Alone or in Combination with Gefitinib

**Figure 8:**
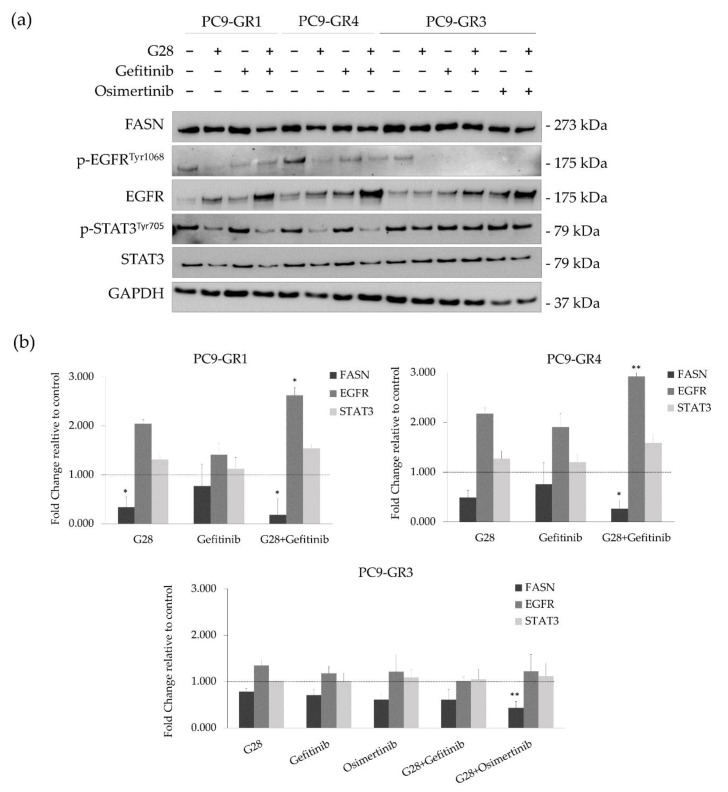
FASN, EGFR, and STAT3 protein and mRNA expression in sensitive (PC9) and Gefitinib Resistant (GR) models (PC9-GR1, PC9-GR3, and PC9-GR4) treated with FASN inhibitors (EGCG and G28) in combination with EGFR TKIs (gefitinib and osimertinib). (**a**) Western blot analysis of FASN, EGFR, and STAT3 in PC9-GR1, PC9-GR3, and PC9-GR4 models treated with G28 alone and in combination with gefitinib, and PC9-GR3 treated with G28 and osimertinib for 72 h. Results shown are representative from three independent experiments. Untreated cells are used as internal control (Ctrl) and GAPDH as a loading control. (**b**) FASN, EGFR, and STAT3 mRNA levels analysis in PC9-GR1, PC9-GR3, and PC9-GR4 models treated with G28 in combination with gefitinib, PC9-GR3 model treated with the combination G28 and osimertinib for 72 h. mRNA levels were obtained by qRT-PCR and normalized against the GAPDH gene. All conditions were compared to the control (untreated cells), which was normalized to 1 (indicated by the dotted line) and expressed as a fold change. Experiments were performed at least three times. * *p* < 0.050, ** *p* < 0.010, *** *p* < 0.001 indicate levels of statistical significance.

In order to discern whether G28 is able to reduce the STAT3 activation produced by EGFR TKIs, a combinatorial analysis was performed (Figure 8). As before, FASN, EGFR, and STAT3 protein and mRNA levels were analyzed using Western blot (Figure 8a) and qRT-PCR (Figure 8b) in GR models treated with synergistic drug concentrations (all of them under the IC_50_ value). The uncropped Western blots can be found in Figure S2c. Therefore, GR models were treated with G28 at 15 µM in combination with 1 µM gefitinib, which is the highest clinically achievable plasma concentration [5]. Osimertinib-resistant PC9-GR3 model was co-treated with 15 µM G28 and 0.5 µM osimertinib. All concentrations were also used in single-treatment to elucidate the effects produced by the combination.

T790M+ GR models treated with G28 alone and in combination with gefitinib showed both FASN protein and mRNA expression decrease. In the PC9-GR3 model, the FASN protein was slightly diminished in mono- and co-treatments. This decrease is in accordance to FASN mRNA expression, which was significantly reduced in combination with osimertinib. Although G28 alone showed more activated EGFR compared to control in all GR models, the combination of G28 with both EGFR TKIs decreased p-EGFR levels. Regarding the total EGFR, co-treatment resulted in higher protein levels compared to monotreatment in all GR models. However, only T790M+ GR models treated with G28 in combination with gefitinib presented significantly higher EGFR mRNA expression (PC9-GR1 *p* = 0.036 and PC9-GR4 *p* = 0.040). Moreover, G28 both alone and in combination with gefitinib reduced STAT3 activation in T790M+ GR models. No changes in STAT3 activation were seen in PC9-GR3 in any treatment. None of the models analyzed showed alterations in the STAT3 protein or mRNA expression levels.

## 3. Discussion

Despite significant advances in EGFRm NSCLC treatment, current therapy is still ineffective to many patients due to the late stage of diagnosis and acquisition of resistance to EGFR TKIs [2,6]. Hence, several efforts have been made on the identification of EGFR TKIs resistance mechanisms to develop an effective treatment for these patients. Some authors pointed out the important role of FASN in drug resistance due to its capacity to allow fast synthesis of new phospholipids for membrane remodeling and plasticity [49,50]. Although the relationship between EGFR and FASN remains unclear, it has recently been described that EGFR upregulates FASN in TKI-resistant EGFRm NSCLC [41]. In addition, FASN inhibition showed cytotoxic effects in lung cancer [40] and resensitized cells to chemotherapy, anti-EGFR and anti-HER2 therapies in breast cancer [38,51]. Therefore, the FASN enzyme may become a promising target for anticancer therapy in EGFRm NSCLC.

Here we studied the effects of natural polyphenolic compound EGCG and its derivative G28 to overcome EGFR TKI resistance in sensitive and GR models. The higher FASN protein levels observed in EGFR TKI resistant models (Figure 1) demonstrated its potential involvement in EGFR TKI resistance acquisition. FASN inhibitors showed similar cytotoxic effects between sensitive and resistant models with IC_50_ values ranging from 75 to 90 µM for EGCG and from 12 to 18 µM for G28 (Figure 3). As determined by K. Jacobsen and coworkers, PC9-GR1 is a T790M+ GR model that also presents MET and EphA2 activation, the PC9-GR3 model exhibits AXL overexpression, and the T790M+ PC9-GR4 model shows EphA2 activation and AXL overexpression [45]. In correlation with this, the higher G28 IC_50_ found in PC9-GR3 model and the significantly similar IC_50_ values in the two T790M+ GR models indicate that none of the known resistance mechanisms described are related to FASN inhibition.

Both compounds have the ability to internalize and inhibit FASN activity as observed in parental PC9 cells (Figure 4), however G28 was of average 5.5 times more effective than EGCG. We have previously shown the ability of the natural compound EGCG to inhibit FASN activity in wild type EGFR NSCLC cells and different breast cancer subtypes [40,43]. Despite their cytotoxicity, only the synthetic compound significantly reduced FASN activity in GR models (Figure 4). Other studies proved the ability of G28 to inhibit FASN activity in triple-negative breast cancer (TNBC) cells [43]. Nevertheless, our study demonstrated that EGCG anticancer activity was independent of FASN inhibition in GR models. It has been extensively described that EGCG has multiple targets and it is involved in multiple signaling pathways and transcription factors, membrane-associated receptors tyrosine kinase (RTKs), or DNA methylation [33]. Some authors exposed that the mechanism underlying EGCG antitumor potency is due to the suppression of the EGFR signaling pathway in NSCLC [52]. Others observed a very stable complex between EGCG and EGFRm (exon 19 ELREA deletion) that was lost with the T790M substitution [53]. Kozue and coworkers showed that EGCG reduced stemness and immunogenicity in EGFR wild type NSCLC cells in vitro and in vivo through the inhibition of AXL [54]. AXL is a tyrosine kinase receptor that has been related to drug resistance and the induction of malignant properties [55] and is overexpressed in GR models [45].

The apoptosis induction was verified by PARP cleavage for all treatments in sensitive and T790M− GR models (Figure 5). However, no PARP cleavage was observed through Western blot analysis in T790M+ GR models after G28 treatment. These results suggest that G28 might cause an apoptosis-independent proliferation reduction in T790M+ GR models as found by others in NSCLC wild-type EGFR models treated with the natural plant polyphenol resveratrol (3,5,40-trihydroxystilben) [56]. The anticancer activity of polyphenols has been shown to be mediated by numerous mechanisms including cell cycle arrest. EGCG, among other natural compounds, showed down-regulation of cyclin-dependent kinases (CDK) and modulation in CDK inhibitors in different human carcinomas [33,57]. The lack of PARP cleavage in some of the treatments and models could be due to the activation of other programmed cell death mechanisms such as autophagy [58].

Alteration in EGFR expression was observed after treatment with FASN inhibitors. EGCG produced a decrease in EGFR protein levels in T790M+ GR models, which is in agreement with Ali et al., who observed an EGFR decrease in sensitive and resistant PC9 cells treated with Orlistat, a FDA-approved FASN inhibitor for obesity management [41]. The synthetic G28 compound increased EGFR mRNA levels in all models, indicating that EGFR overexpression could be an alternative pathway to FASN inhibition. The EGFR activation after FASN gene overexpression in epithelial breast cells has been previously shown [42]. Furthermore, our results suggest that the EGFR pathway could be implicated in FASN regulation at a transcriptional or translational level as exposed before [40]. Despite all models increased EGFR mRNA expression after G28 treatment, only PC9 cells seemed to compensate the effect of G28 by increasing EGFR activation. Therefore, cell proliferation reduction found in all models after FASN inhibition (Figure 3) could be due to the lack of post-translational palmitoylation substrate [41].

It seems increasingly clear that persistent STAT3 activation is related to EGFR-based therapies resistance [13]. STAT3 is under the control of different cytokines and growth factors playing an important role in metastasis, proliferation, survival, invasion, migration, and angiogenesis [14,15,16]. Natural polyphenols, normally multitarget inhibitors, are now emerging as promising STAT3 inhibitors or its upstream signaling molecules Src, gp130, or NFкB [18]. Among them, EGCG has been reported to reduce STAT3 phosphorylation in head and neck carcinomas [59] and pancreatic cancer [60]. Based on the above, we hypothesized that EGCG and its derivative G28 would diminish the STAT3 activation produced by EGFR TKIs in NSCLC cells. Both compounds reduced p-STAT3 levels in a dose-dependent manner when used alone in comparison to control samples. In agreement with previous studies, STAT3 was activated by EGFR TKIs (Figure 6) [22].

Combinatorial treatments of EGCG and G28 with EGFR TKIs were performed in order to study their effects on GR models (Figure S1). The combination of gefitinib and osimertinib with EGCG showed additivism whereas synergistic effects were identified in combination with G28 (Figure 7). Other authors have previously reported synergistic outcomes after co-treatment with a STAT3 inhibitor (TPCA-1) and EGFR TKI in EGFRm NSCLC models [61]. Previous results from our group demonstrated that G28 compound had a synergistic interaction with pertuzumab and temsirolimus in HER2+ breast cancer cells [38] and EGCG with cetuximab in TNBC [51]. Interestingly, G28 in combination with gefitinib decreased the activation of STAT3 to the same extent as when used alone in T790M+ GR models. Thus, other mechanisms must be involved in the synergistic effects found. On the other hand, no differences in p-STAT3 were observed in PC9-GR3. This could be, in part, due to the multiple pathways that can be altered in the acquisition of gefitinib and osimertinib double-resistance in PC9-GR3 model such as mutations in EGFR/STAT3 or other related up- or downstream signaling molecules, leading to the constitutive activation of STAT3. The analysis of the main genes regulated by the STAT3 transcription factor could provide relevant information to identify some pathways alterations after FASN inhibitors treatment. The synergistic effects found in GR models co-treated with FASN inhibitor G28 and EGFR TKIs supports the idea that EGFR palmitoylation mediated by FASN leads to TKI resistance acquisition in EGFRm NSCLC [41]. However, the specific G28 mechanism of action and possible targets are still unknown and further studies are needed. Wu et al. suggested that FASN inhibition may cause an imbalance in the membrane lipids levels, which may produce a membrane localization decrease of IGF-1R, and the inactivation of the downstream STAT3 signaling pathway [36]. Furthermore, the IGF-1R is a transmembrane tyrosine kinase linked to MAPK and PI3K/AKT pathways, shared with EGFR, which could explain the effects found only in T790M+ GR models [62].

Taken together, our observations suggest that FASN has a key role in acquired TKI-resistant EGFRm NSCLC. The inhibition of this enzyme resensitizes cells to EGFR TKIs treatments. These results encourage for further studies to analyze the combinatorial treatment of FASN inhibitors and EGFR TKIs to overcome the EGFR TKI resistance in NSCLC. 

## 4. Materials and Methods

### 4.1. Cell Lines and Culture Conditions

Human adenocarcinoma PC9 cells and its gefitinib resistant derivatives PC9-GR1, PC9-GR3, and PC9-GR4 models [45] were kindly provided by Dr. R. Rosell and Dr. M. A. Molina (Barcelona, Spain). All cells were routinely grown in RPMI-1640 medium (Lonza, Basel, Switzerland), supplemented with 10% fetal bovine serum (FBS; HyClone Laboratories, GE Healthcare, Chicago, IL, USA), 50 U/mL penicillin, and 50 µg/mL streptomycin (Lonza). In all cases, the cells were used immediately after resuscitation and were maintained at 37 °C in a humidified atmosphere with 5% CO_2_, propagated following established protocols, remaining free of mycoplasma throughout the experiments.

### 4.2. Cell Proliferation Assays

To investigate the effect of EGFR TKIs or FASN inhibitors cells were seeded into 96-well plates at the appropriate density in their growth medium. Gefitinib and osimertinib were kindly provided by AstraZeneca. EGCG was purchased from Sigma (USA) and G28 was synthesized as described elsewhere [43]. For monotreatment assays, after 24 h cells were treated with different concentrations of each drug for 72 h. Cell viability was determined by the 3-(4,5-dimethyl-2-thiazolyl)-2,5-diphenyl-tetrazolium bromide (MTT) assay (Sigma) as described elsewhere [44]. For drug combination experiments, cells were treated with three fixed concentrations of gefitinib (1, 2.5, and 5 µM) or osimertinib (0.5, 1, and 2 µM) in combination of a series of increasing concentrations of EGCG or G28 for 72 h. Following treatment, cell proliferation was measured using the standard colorimetric MTT assay. Using the multi-well plate reader Benchmark Plus (Bio-Rad Laboratories, Inc., CA, USA), absorbance was determined at 570 nm. Combinatorial effects were evaluated using the combination index (CI) based on the Chou and Talalay method [48] using the CompuSyn^TM^ software (Biosoft, MO, USA). CompuSyn^TM^ calculates the CI values; if the value equals 1 the effect is considered additive, if above 1 antagonistic and below 1 synergistic. Data presented are from three separate wells per assay, and the assay was performed at least three times.

### 4.3. Western Blot Analysis of Cell Lysates

Gefitinib-sensitive and -resistant models were treated with EGFR TKI, FASN inhibitor, or the combination of both drugs for 72 h. Afterwards, attached and floating cells were harvested and lysed in ice-cold lysis buffer (Cell Signaling Technology Inc., MA, USA) containing 100 µg/mL phenylmethylsulfonylfluoride (PMSF; Sigma) by vortexing every 5 min for 30 min. Protein concentration was determined by the Lowry method (DC Protein Assay, Bio-Rad Laboratories, Inc.). Equal amounts of protein were heated in lithium dodecyl sulfate (LDS) sample buffer with a sample reducing agent (Invitrogen, CA, USA) for 10 min at 70 °C, separated by SDS-PAGE and transferred to nitrocellulose membranes (ThermoFisher Scientific Inc., MA, USA). Membranes were incubated for 1 h at room temperature in blocking buffer (5% skim milk powder in Tris-buffered saline (TBS)) 0.05% Tween (TBS-T) and overnight at 4 °C with the following primary antibodies diluted in blocking buffer: FASN (Cell Signaling Technology Inc.; #3180), p-STAT3^Tyr705^ (Cell Signaling Technology Inc.; #9131), p-EGFR^Tyr1068^ (Cell Signaling Technology Inc.; #2234), PARP (Cell Signaling Technology Inc.; #9542), EGFR (Cell Signaling Technology Inc.; #2232), STAT3 (Cell Signaling Technology Inc.; #4904), and GAPDH (Proteintech, Manchester, UK; #60004-1-IG). Specific horseradish peroxidase (HRP)-conjugated secondary antibodies were incubated for 1 h at room temperature. The immune complexes were detected using chemiluminescent HRP substrate Clarity^TM^ Western ECL Substrate (Bio-Rad Laboratories, Inc.) or West Femto Maximum Sensitivity Substrate (ThermoFisher Scientific Inc.) in a Bio-Rad ChemiDoc^TM^ MP Imaging System (Bio-Rad Laboratories, Inc.). Western blot analyses were repeated at least three times and representative results are shown. 

### 4.4. Quantitative Real-Time PCR (qRT-PCR) Analysis

Cells were treated with EGFR TKIs or FASN inhibitors as a single agent or in combination for 72 h. Then, cells were PBS washed and resuspended in 1 mL of Qiazol (Qiagen, Hilden, Germany). GeneJET RNA Purification Kit (ThermoFisher Scientific Inc.) was used to isolate total RNA following the manufacturer’s instructions. RNA samples were quantified using a Nano-Drop 2000 Spectophotometer (ThermoFisher Scientific). Total RNA was reverse-transcribed into complementary DNA (cDNA) using a High Capacity cDNA Archive Kit (Applied Biosystems, CA, USA). Different gene expression levels were determined using QuantStudio3 Real-time PCR System (ThermoFisher Scientific Inc.) with qPCRBIO SyGreen Mix Lo-Rox real-time PCR (PCR Biosystems Inc., PA, USA), following manufacture instructions. Primers used are shown in Table 1. qRT-PCR analyses were performed at least four times and each gene was run in triplicate. Gene expression levels were quantified using the standard formula 2^^dCT^ and normalized to the housekeeping GAPDH (2^^dCT^).

### 4.5. Inhibition of Fatty Acid Synthase Activity

Cells were seeded in 24-well plates at a density of 3 × 10^4^ cells/well in RPMI supplemented with 10% fetal bovine serum (FBS). 24 h later, the maintenance medium was replaced by the treatment medium (RPMI-1640 supplemented with 1% lipoprotein-deficient FBS (Sigma-Aldrich)) along with the assayed compounds or vehicle (dimethyl sulfoxide, DMSO, Sigma-Aldrich). Cells were treated with a concentration corresponding to the previously determined IC_50_ for 72 h. For the last 6 h (1,2-14C) acetic acid sodium salt (53.9 mCi/mmol, Perkin Elmer Biosciences, Waltham, MA, USA) was added to the medium (0.5 µCi/mL). The lipid extraction was performed as previously described [43]. Cells were washed twice with phosphate-buffered saline (PBS, HyClone Laboratories, Logan, UT, USA) and once with MeOH/PBS (2:3). Cell pellets were resuspended in 0.2 M NaCl and lysed with freeze (liquid N_2_)—thaw (37 °C) cycles. Then, lipids were extracted with CHCl_3_/MeOH (2:1) and 0.1 M KOH. The organic phase was washed with CHCl_3_/MeOH/H_2_O (3:48:47) and the solvents were evaporated under vacuum conditions. Finally, pellets were resuspended in EtOH and counted by scintillation. The total protein content was quantified by the Bradford assay (Sigma-Aldrich).

### 4.6. Statistical Analysis

Parametric data were analyzed by the Student’s *t* test when comparing two groups or the one-way analysis of variance (ANOVA) followed by Bonferroni or Tamhane’s T2 post hoc test for multiple comparisons. The non-parametric data were analyzed with the Mann–Whitney U tests for non-normally independent variables; otherwise, the Kruskal–Wallis test was used for more than two groups. All data are expressed as mean ± SE. Levels of significance were set at *p* < 0.050 and are represented by asterisks, as follows: *p* < 0.050 (denoted as *), *p* < 0.010 (denoted as **), and *p* < 0.001 (denoted as ***). The statistical analysis was performed using the IBM SPSS software (Version 21.0; SPSS Inc., IL, USA).

## 5. Conclusions

The need to find more effective and less toxic therapeutic treatments for EGFRm NSCLC patients is one of the major challenges in lung cancer research. Natural compounds are emerging as potential anticancer candidates for their safety and multitarget intrinsic features. Improvement of the properties of natural compounds through the design of synthetic derivatives is aimed at maintaining these features while increasing, efficacy, bioavailability, and stability in physiological conditions.

Here we show additive and synergistic effects of the polyphenolic plant-derived EGCG compound and its derivative G28, respectively, in combination with EGFR TKIs in GR models. Despite the exact mechanism by which these compounds are cytotoxic is still unknown, our results shed light on their ability to modulate FASN/EGFR/STAT3 pathways. The capacity to affect multiple pathways might prove useful in overcoming other drug resistances. Further analyses are required to completely understand the mechanism of action.

Taken together, this paper supports the inhibition of the metabolic enzyme FASN by G28 compound in combination with EGFR TKIs as a new potential strategy for resistant EGFRm NSCLC.

## Figures and Tables

**Table 1:** Primer design.

FASN	Forward	CAGGCACACACGATGGAC
	Reverse	CGGAGTGAATCTGGGTTGAT
STAT3	Forward	CACCTTCAGGATGTCCGGAA
	Reverse	ATCCTGGAGATTCTCTACCACTTTCA
EGFR7	Forward	CATGTCGATGGACTTCCAGA
	Reverse	GGGACAGCTTGGATCACACT
GAPDH	Forward	TCTTCCAGGAGCGAGATC
	Reverse	CAGAGATGATGACCCTTTTG

## Supplementary Materials

The following are available online at https://www.mdpi.com/2072-6694/12/5/1283/s1, Figure S1: Proliferative curves of FASN inhibitors (EGCG and G28) alone and in combination with EGFR TKIs (gefitinib and osimertinib) in EGFR TKIs resistant models, Figure S2. Whole Western blot figures showing PARP, FASN, EGFR, STAT3 and GAPDH protein bands with molecular weight markers (merge of colorimetric and chemiluminescence). (a) Western blot images from Figure 5. (b) Western blot images from Figure 6. (c) Western blot images from Figure 8.
