# Supplementary material for: Fatty Acid Synthase Inhibitor G28 Shows Anticancer Activity in EGFR Tyrosine Kinase Inhibitor Resistant Lung Adenocarcinoma Models
Source: Cancers (Basel). 2020 May 19;12(5):1283. doi: 10.3390/cancers12051283 (PMC7281741; doi:10.3390/cancers12051283)
Supplement: Supplementary file 1 [file cancers-12-01283-s001.pdf]

Fatty Acid Synthase Inhibitor G28 Shows Anticancer Activity in EGFR Tyrosine Kinase Inhibitor Resistant Lung Adenocarcinoma Models

Emma Polonio-Alcalá, Sònia Palomeras, Daniel Torres-Oteros, Joana Relat, Marta Planas, Lidia Feliu, Joaquim Ciurana, Santiago Ruiz-Martínez and Teresa Puig

(a)

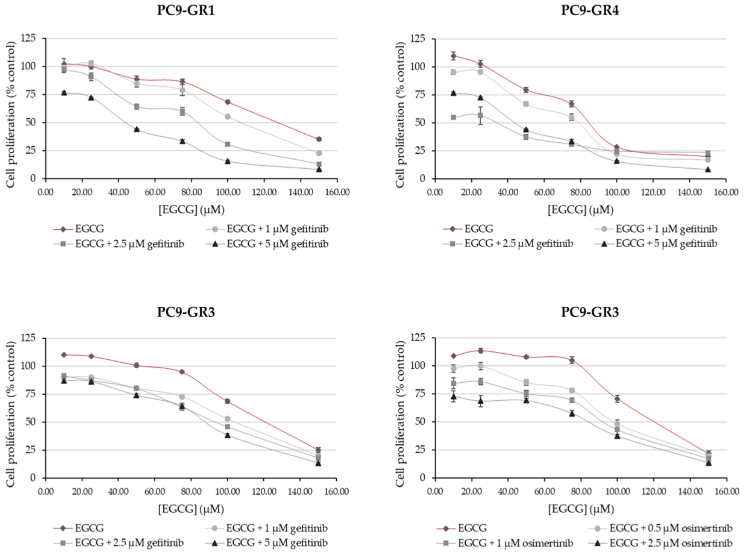

(b)

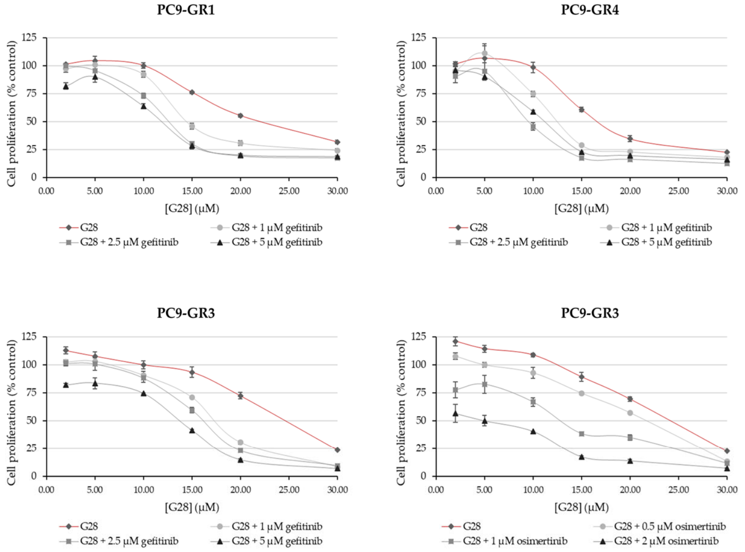

Figure S1. Proliferative curves of FASN inhibitors (EGCG and G28) alone and in combination with EGFR TKIs (gefitinib and osimertinib) in EGFR TKIs resistant models. (a) EGCG (10-150 μM) alone and in combination with gefitinib (1, 2.5 and 5 μM) in PC9-GR1, PC9-GR3, and PC9-GR4 models and osimertinib (0.5, 1 and 2 μM) in PC9-GR3. (b) G28 (2-30 μM) alone and in combination with gefitinib (1, 2.5 and 5 μM) in PC9-GR1, PC9-GR3, and PC9-GR4 models and osimertinib (0.5, 1 and 2 μM) in PC9-GR3. Results were determined using the MTT assay for 72h. Experiments were performed at least three times. Results shown are mean ± SE.

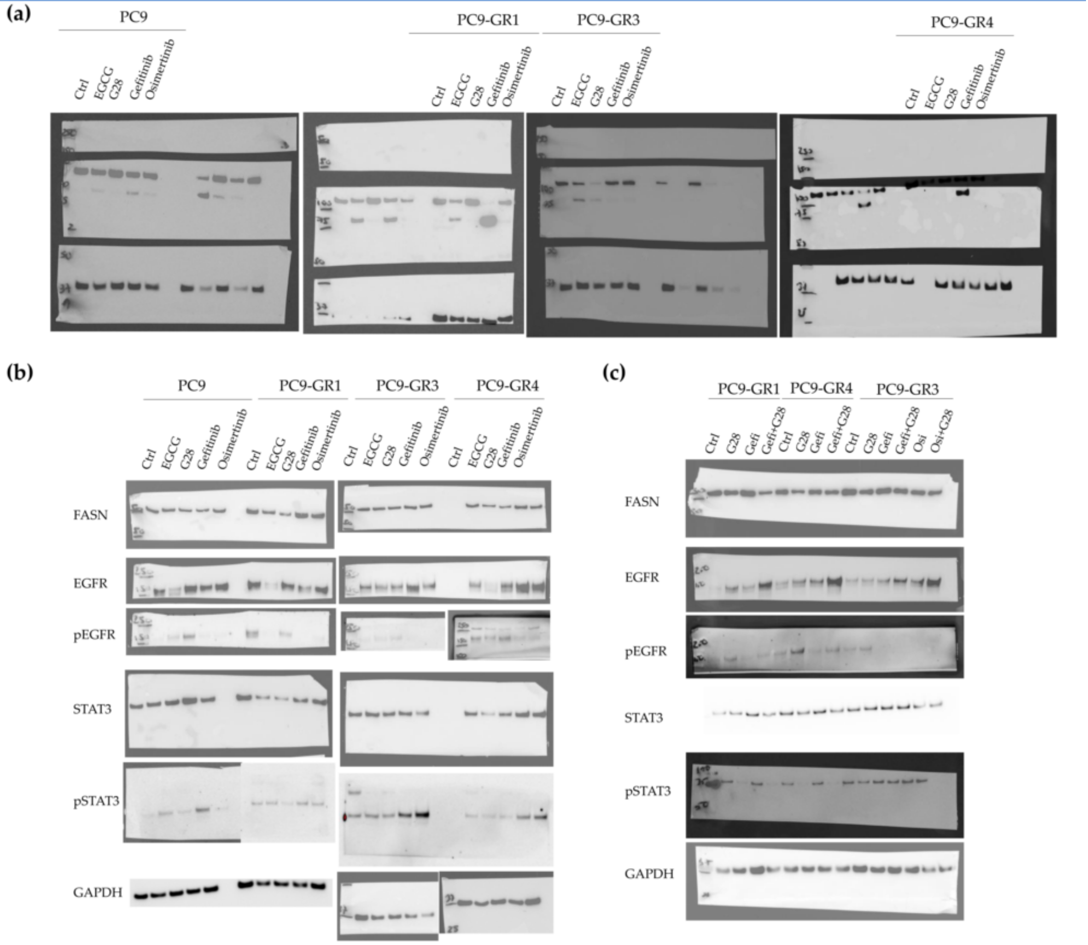

Figure S2. Whole Western blot figures showing PARP, FASN, EGFR, STAT3 and GAPDH protein bands with molecular weight markers (merge of colorimetric and chemiluminescence). (a) Western blot images from Figure 5. (b) Western blot images from Figure 6. (c) Western blot images from Figure 8.
